# Contraction Phase and Force Differentially Change Motor Evoked Potential Recruitment Slope and Interhemispheric Inhibition in Young Versus Old

**DOI:** 10.3389/fnhum.2020.581008

**Published:** 2020-10-06

**Authors:** Elsa Ermer, Stacey Harcum, Jaime Lush, Laurence S. Magder, Jill Whitall, George F. Wittenberg, Michael A. Dimyan

**Affiliations:** ^1^University of Maryland, Baltimore, MD, United States; ^2^Department of Neurology, School of Medicine, University of Maryland, Baltimore, Baltimore, MD, United States; ^3^Department of Epidemiology and Public Health, School of Medicine, University of Maryland, Baltimore, Baltimore, MD, United States; ^4^Department of Physical Therapy and Rehabilitation Science, School of Medicine, University of Maryland, Baltimore, Baltimore, MD, United States

**Keywords:** cortical dynamics, aging, disinhibition, interhemispheric inhibition (IHI), motor evoked potential (MEP) recruitment slope

## Abstract

Interhemispheric interactions are important for arm coordination and hemispheric specialization. Unilateral voluntary static contraction is known to increase bilateral corticospinal motor evoked potential (MEP) amplitude. It is unknown how increasing and decreasing contraction affect the opposite limb. Since dynamic muscle contraction is more ecologically relevant to daily activities, we studied MEP recruitment using a novel method and short interval interhemispheric inhibition (IHI) from active to resting hemisphere at 4 phases of contralateral ECR contraction: Rest, Ramp Up [increasing at 25% of maximum voluntary contraction (MVC)], Execution (tonic at 50% MVC), and Ramp Down (relaxation at 25% MVC) in 42 healthy adults. We analyzed the linear portion of resting extensor carpi radialis (ECR) MEP recruitment by stimulating at multiple intensities and comparing slopes, expressed as mV per TMS stimulation level, via linear mixed modeling. In younger participants (age ≤ 30), resting ECR MEP recruitment slopes were significantly and equally larger both at Ramp Up (slope increase = 0.047, *p* < 0.001) and Ramp Down (slope increase = 0.031, *p* < 0.001) compared to rest, despite opposite directions of force change. In contrast, Active ECR MEP recruitment slopes were larger in Ramp Down than all other phases (Rest:0.184, *p* < 0.001; Ramp Up:0.128, *p* = 0.001; Execution: *p* = 0.003). Older (age ≥ 60) participants’ resting MEP recruitment slope was higher than younger participants across all phases. IHI did not reduce MEP recruitment slope equally in old compared to young. In conclusion, our data indicate that MEP recruitment slope in the resting limb is affected by the homologous active limb contraction force, irrespective of the direction of force change. The active arm MEP recruitment slope, in contrast, remains relatively unaffected. Older participants had steeper MEP recruitment slopes and less interhemispheric inhibition compared to younger participants.

## Introduction

Force production in limb muscles increases corticospinal motor evoked potential (MEP) amplitudes in resting contralateral homolog muscles (Ferbert et al., 1992), an effect likely mediated by transcallosal circuits (Perez and Cohen, 2009). This effect is missing in stroke-affected corticospinal tract, and its presence is correlated with greater recovery from post-stroke hemiparesis (Dimyan et al., 2014). However, the rise and fall of force from muscle contraction is not instantaneous. Especially in day-to-day tasks, gradual increases and decreases in muscle activity predominate compared to ballistic muscle contractions and sudden reductions in force. Despite this feature, little is known about how the development of a forceful muscle contraction (or its relaxation) drives contralateral corticospinal MEP recruitment. Because it is known that corticospinal drive to the active arm is greater during development of force compared to reduction of force even at equivalent torque production (Kimura et al., 2003), it is reasonable to hypothesize that these differences may be communicated to the opposite hemisphere. To better understand the interplay between motor cortices, we sought to examine the corticospinal MEP recruitment slope in the resting limb during different **phases of muscle contraction**, i.e., during the **ramp-up** in muscle activity from rest to target and during the **ramp-down** from target to rest. We hypothesized that the **ramp-up** and **ramp-down** phases of isometric muscle contraction, despite reaching similar levels of force at their force-production midpoints, differ in corticospinal drive. Elucidating the effects of force versus phase of muscle contraction on contralateral corticospinal drive has implications for interhemispheric mechanisms of motor control and rehabilitation after unilateral stroke.

Aging is associated with reduced functioning of arm coordination that leads to reduction in activities of daily living such as dressing, preparing meals, and engaging in work and leisure activities (Woytowicz et al., 2016). Increasing evidence points to a major role for neurophysiological mechanisms of motor control in this decline, along with age-related cognitive and neuromuscular alterations (McGinley et al., 2010; Coppi et al., 2014; Levin et al., 2014; Papegaaij et al., 2014; Fujiyama et al., 2016). Interhemispheric inhibition (IHI) is a broad term used to describe the inhibitory influence of each hemisphere’s primary (M1) and premotor cortices on the contralateral primary motor cortex (Boddington and Reynolds, 2017) and there are likely multiple separate circuits that can contribute to some form of IHI. IHI is thought to contribute to prevention of mirror movements, and release of premotor-motor IHI may be a compensatory mechanism to offset age-related slowing of ballistic finger movements (Hinder et al., 2010; Hinder, 2012). Thus, the IHI circuit is one of the interhemispheric circuits that may be important for unilateral MEP recruitment slope, and we sought to determine whether IHI from the active to resting limb is modulated similarly compared to MEP recruitment slope. At rest, IHI may be reduced in older age, leading to increased cortical activity and potentially to reduced focus of neural representation (Fujiyama et al., 2009; Fling and Seidler, 2011; Fling et al., 2011). These neurophysiological findings may be the mechanistic source of age-related changes in motor behavior such as increased motor overflow and mirror movements (Addamo et al., 2007; Cincotta and Ziemann, 2008; Koerte et al., 2010).

The goal of the experiments described here was to measure changes in MEP recruitment slopes in the resting non-dominant arm of healthy participants while they performed each phase of isometric muscle contraction with the dominant arm. We used a novel method to efficiently account for inter-subject variability in stimulation intensities for threshold and plateau levels of MEP recruitment. In addition to examining these changes, we aimed to determine how short-interval IHI from active to resting M1 (just one of the forms of non-invasively testable modulatory motor circuits) was modulated by unilateral muscle contractions and whether aging interacted with either phenomenon. We hypothesized that resting non-dominant arm MEP recruitment slope would be modulated as a function of dominant arm force production and that older age would reduce both MEP recruitment slope and IHI.

## Materials and Methods

### Ethical Approval

This study was approved by the Institutional Review Board of the University of Maryland, Baltimore, under the standards of the latest version of the Declaration of Helsinki, except for registration in a publicly available database, which was not required at the time of study onset.

### Participants

Fifty-one healthy adult volunteers provided written informed consent to participate in this study and met all inclusion and no exclusion criteria. Specifically, participants were included if they scored as right-handed on the Edinburgh Handedness Inventory (≥+40) (Oldfield, 1971), scored in the normal range (≥26) on the Montreal Cognitive Assessment (MOCA) (Nasreddine et al., 2005), had no history of seizure within 10 years prior to enrollment and were using no medications to prevent seizures, were taking no central nervous system stimulant medications or illicit drugs, had no significant upper extremity injuries, had normal upper extremity strength and sensation on examination, and had no history or presence of neurological diagnosis aside from headaches as assessed by a medical history and physical exam (Rossetti et al., 2011; Nasreddine et al., 2012).

### Electromyography Recording

Surface electromyography (sEMG) activity was recorded from electrodes placed on the skin over the bellies of three muscles in each arm: extensor carpi radialis, flexor carpi radialis, and biceps brachii using active electrodes with integrated ground (B&L Engineering, Santa Ana, CA, United States). Electrode placement was determined following principles of sEMG (Cram et al., 1998) and individual participant muscle palpation. The sEMG signal was collected at 5000 Hz and was amplified (gain = 330) and channeled through a digital/analog converter for visualization, recording, and analysis on a PC [Power1401mkII DAC & Signal Software v6 (RRID:SCR_017081), Cambridge Electronic Design, Ltd., Cambridge, United Kingdom]. The wrist force task required isometric activation of the right extensor carpi radialis (R-ECR) in the active arm. The target muscle of interest was the left extensor carpi radialis (L-ECR) in the resting arm. EMG of both arms was monitored for background voluntary activity when the participant was meant to be at rest. When this occurred, the participant was given the chance to relax the muscle, and the session was resumed. Background EMG activity was recorded and used for *post hoc* analysis as explained later (see section “L-ECR Model Development”).

### Transcranial Magnetic Stimulation

#### Hotspot Determination

Transcranial magnetic stimulation (TMS) of the motor cortex was performed using custom fabricated 60 mm diameter Double Coils and two MagStim 200^2^ Magnetic Stimulators and (MagStim Ltd., Wales, United Kingdom) controlled by computer interface. The hotspot of the muscle of interest, ECR, was identified as the coil location on the scalp that evoked the largest MEP amplitude using the lowest stimulation intensity with coil-handle angled posteriorly and 45 degrees from the sagittal line (Rossini et al., 1994). The hotspot for ECR on each side was determined, digitized, and recorded on a computer using infrared motion tracking via the BrainSight system on the MNI152 averaged brain template (Rogue Research, Montreal, Canada), allowing us to precisely maintain the TMS coils in the correct positions throughout the experiment. Once hotspots were determined for ECR for each hemisphere, if both coils did not fit on the participant’s head simultaneously due to interference of the coil wings with each other, the handle of the coil targeting left primary motor cortex was rotated to 90 degrees from the sagittal line. This accommodation allows both coils to fit on an individual participant’s head since the conditioning stimulus for short-interval IHI has been determined to be independent of coil yaw (Chen et al., 2003). This was done while still maintaining the same hotspot location. If such an adjustment was made, the hotspot was confirmed at this new angle by testing scalp locations in a 0.5 cm grid around the original hotspot. In no participants did a new hotspot have to be generated due to a change in handle direction. The coil yaw had to be adjusted for 50% of younger participants and 80% of older participants to allow two coils to fit on the head simultaneously.

#### MEP Recruitment Sampling

The choice of methods to measure MEPs efficiently in different groups is critical in order to avoid ceiling and floor effects (Moller et al., 2009; Cuypers et al., 2014), control for contralateral confounding activity (Daskalakis et al., 2002), and account for the spatial reduction in muscle representation that can occur with aging (Coppi et al., 2014). We developed a unique method that would sample the linear portion of each individual’s MEP recruitment most efficiently. Since it is difficult to know at which level a participant’s MEP recruitment will begin its plateau, we needed a surrogate marker to more efficiently find this intensity. Given that there is a spatial separation between muscle representations in the cortex (Meier et al., 2008; Cuypers et al., 2014; Kukke et al., 2014), we hypothesized that by limiting our range of stimulating intensities based on activation of more proximal muscle representation, we would focus our sampling of MEP amplitudes to the most linear portion of recruitment and avoid the plateau. We chose to sample from the resting motor threshold of ECR to the resting motor threshold of Biceps Brachii in order to achieve this. Since this would lead to different stimulation intensities in different participants, we used the linear slopes of the sampled recruitment to assess corticospinal drive across participants (Liuzzi et al., 2013; Kukke et al., 2014). In a sample of *n* = 10 participants, we compared our novel method of recruitment curve sampling to the more traditional method of rMT intervals and demonstrated that our method achieved its goal of sampling at the linear portion of the recruitment curve (see Supplementary Material). This method also allowed us to statistically control for covariates (such as right arm MEP, and background EMG) at each phase of muscle contraction rather than experimentally attempting to control them.

We started with the test stimulus (TS) hemisphere (targeting resting L-ECR representation in right primary motor cortex) and identified the resting motor threshold (rMT) determined as the minimum stimulation intensity needed at the hotspot to evoke motor potentials greater than or equal to 0.05 mV on five out of ten trials (Rothwell et al., 1999) in the L-ECR. Next, the maximum stimulus intensity (MAX) for the ECR recruitment curve was determined as the minimum stimulation intensity at the ECR hotspot to evoke MEP in the biceps muscle greater than or equal to 0.05 mV on 5 out of 10 trials, with the constraint that the MAX be at least 8% Maximum Stimulator Output (MSO) higher than the ECR rMT to allow for an adequate MEP recruitment slope measurement. The range of rMT to MAX was divided into eight equal intervals. The TS MEP recruitment slope was obtained by stimulating at 8 intensities from 1 interval below rMT up to MAX. This procedure was repeated for the conditioning stimulus (CS) side (targeting active R-ECR representation in left primary motor cortex), except that only three stimulus intensities were used ranging from one interval above rMT to one below MAX. The values of rMT and MAX did not significantly differ across age groups (see Table 1).

**TABLE 1:** Characteristics for younger and older participants, means (and standard deviations).

Measure		Younger	Older
*n*		22	20
Sex	# Female	14	9
Age	years	25.0 (2.71)	68.1 (5.25)
	Range, years	22–30	60–80
Edinburgh Handedness Score	≥+40	80 (13.0)	81 (15.7)
Montreal Cognitive Assessment	≥26	29 (1.44)	28 (1.31)
Nine Hole Peg Test	Dominant (Right), seconds	16.5 (2.23)	20.4 (4.52)
	Dominance Ratio [(1-(D/ND))*100]	8.8 (7.46)	7.6 (8.44)
Maximum Voluntary Contraction Force (Wrist)	Dominant (Right), Nm	7.8 (4.10)	5.4 (3.43)
	Ratio (D:ND)	1.2 (0.33)	1.2 (1.04)
Perceived Effort During Task	% of maximum force	52 (18.4)	46 (25.5)
Resting Motor Threshold (rMT)	TS, MSO%	45 (13.1)	41 (7.4)
	CS, MSO%	47 (9.8)	44 (9.0)
Maximum Stimulus Intensity (MAX)	TS, MSO%	70 (20.4)	60 (13.4)
	CS, MSO%	76 (20.2)	63 (14.9)

*For perceived effort during task ratings, *n* = 12 and *n* = 9 for younger and older participants, respectively. TS, test stimulus; CS, conditioning stimulus.*

For all conditions, three MEP samples were taken at each stimulus intensity since our primary outcome was the linear slope of recruitment, expressed as mV per TMS stimulation level, rather than the average MEP amplitude. Others have demonstrated that only 2 MEP samples are required at each stimulus intensity when modeling MEP recruitment (Kukke et al., 2014), and we confirmed in a separate experiment high correlation between recruitment measured with 10 samples versus 3 samples per stimulus intensity (Cronbach’s α = 0.96, unpublished data). During REST trials, single or paired pulse TMS was delivered at an interstimulus interval of 5 s with 25% jitter between trials. During the active trials, TMS was triggered by the force being produced. Hence interstimulus interval varied with the speed of the participant’s isometric contractions but were no more frequent than once every 5 s.

In 4 younger subjects, 100% MSO was reached before an adequate number of MEP in biceps brachii were identified on both the TS and CS sides. In an additional 4 younger subjects, 100% MSO was reached on the CS side only. In these participants, the MEP recruitment slope was obtained by using 100% MSO for MAX as the highest intensity stimulus in the range. Four subjects received a stimulation range at the minimum 8% MSO interval: one older subject on the TS side and two older subjects and one younger subject on the CS side.

#### Short-Interval Interhemispheric Inhibition

Short-interval IHI is a non-invasive physiologic measure of interaction between the two primary motor cortices. It is demonstrated by delivering a suprathreshold transcranial magnetic stimulus to one primary motor cortex between 6 and 30 ms before delivery of a suprathreshold stimulus to the opposite M1 (Ferbert et al., 1992). The first “conditioning” stimulus (CS), besides activating the corticospinal tract, is believed to stimulate transcallosal fibers which then activate inhibitory interneurons in the opposite M1 and reduces the amplitude of motor-evoked potentials (MEP) elicited by the second “test” stimulus (TS) (Di Lazzaro et al., 1999).

### Experimental Design

The resting non-dominant left arm was placed in a cushioned arm rest with the wrist in neutral position, symmetric to the right arm. The active dominant right arm was placed in a custom arm rest with soft restraints with the dorsum of the hand positioned against a force transducer connected to a six-degrees-of-freedom load cell. The device was designed to isolate wrist kinetics from more proximal arm movements (Hidler et al., 2006) and measure isometric wrist extension force. Prior to the experiment, we established a maximum voluntary isometric contraction force (MVC) for each participant by averaging 3 trials of isometric maximal wrist extension force. Participants also completed the Nine-Hole Peg Test (NHPT) (Mathiowetz et al., 2016) with each arm to establish baseline performance differences in a study visit prior to the experiment.

**FIGURE 1:**
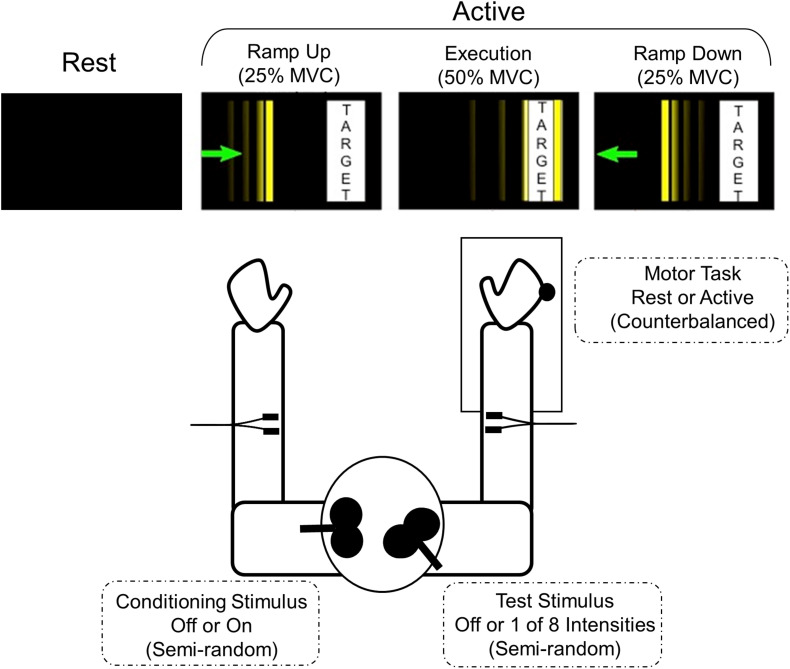
Motor task and experimental set-up. The participant is seated with their shoulders in approximately neutral flexion, 20° abduction, and elbows at approximately 90° flexion. The forearm and wrist are in neutral position bilaterally. The right arm is placed in a custom arm rest with soft restraints with the dorsum of the hand positioned against a force transducer connected to a six-degrees-of-freedom load cell. The visual display for each active phase is depicted. The thin vertical line is the cursor that subjects moved by applying isometric wrist extension force to the joystick. The goal is to move the cursor smoothly toward the target bar, hold within the target bar for at least 1.5 s, and make a controlled relaxation back to rest. The width and position of the target bar are scaled according to the participant’s maximum voluntary contraction (MVC).

After establishing the MVC, the participants were introduced to the task they would be performing during the active trials of the experiment. The force transducer was connected to a computer that both recorded the wrist extension forces produced and translated them to movement of a vertical cursor horizontally across the screen of a monitor. The goal of the active trials was to move the cursor to a target bar whose position and width were scaled to 50% of each participant’s maximum voluntary contraction (MVC) force (see Figure 1). Participants were instructed that at the start of each trial they were to move the cursor at a comfortable pace to the target box (RAMP UP), hold the cursor in the target continuously for 1.5 s (EXECUTION), and perform a controlled relaxation to bring the cursor back to the starting position (RAMP DOWN) and not to simply stop all muscle activity suddenly. Participants completed practice trials until they were comfortable with the task and could perform a controlled relaxation. To determine if perception of effort differed between groups, participants were asked to estimate how much force (as a percent of their MVC) was required to do the task.

Participants performed a total of 420 trials to allow for 3 MEP samples per stimulation state and behavioral condition. Peak-to-peak MEP amplitudes were calculated from a 50 ms window beginning 15 ms after the last TMS pulse. In each trial, one MEP sample was obtained from one of 35 stimulation states, 11 single pulse stimulation states (8TS + 3CS individually) or 24 paired-pulse stimulation states (8TS ^∗^ 3CS), during one of four phases of right arm isometric wrist extensor contraction. As has previously been demonstrated, increasing CS intensities lead to increased inhibition (Ferbert et al., 1992), so for the analysis presented here we compared only absence of CS vs. presence of CS at the highest intensity. The REST phase was sampled with both arms completely at rest, and the three active phases were sampled as follows: RAMP UP: at 25% MVC with force increasing; EXECUTION: after 250 ms at 50% tonic MVC (target force); and RAMP DOWN: at 25% MVC during controlled relaxation with force decreasing. The force transducer was connected to a computer using Simulink software which detected the force levels and communicated with the CED Power1401 DAC to trigger the magnetic stimulation at the precise and appropriate force levels. The stimulation state trials were in a fixed semi-random order, counterbalanced across the three active phases (3 phases ^∗^ 35 stimulation states ^∗^ 3 samples = 315 trials), plus three samples of each stimulation state taken during the Rest phase (35 stimulation states ^∗^ 3 samples = 105 trials). The three samples were averaged for each subject to produce an average MEP for every combination of stimulation state and each of the four phases. All rest trials were performed together, all active trials were performed together, and the order of active trials and rest trials was counterbalanced across subjects (see Figure 1).

### Statistical Analysis

All statistical analyses were performed using IBM SPSS Statistics for Windows Version 25 (SPSS, RRID:SCR_002865). The primary outcome measure, L-ECR MEP recruitment slopes, were analyzed with linear mixed-effects modeling (Gueorguieva and Krystal, 2004; Heise et al., 2013). R-ECR MEP recruitment slopes were similarly analyzed with linear mixed-effects modeling. Planned comparisons for MEP recruitment slopes bilaterally, background EMG bilaterally, and R-ECR MEP amplitudes were used to compare event phases via *t*-test. Demographic and stimulation parameters were compared between younger and older subjects using *t*-tests. Correlations among continuous variables were examined using Pearson’s *r*.

**FIGURE 2:**
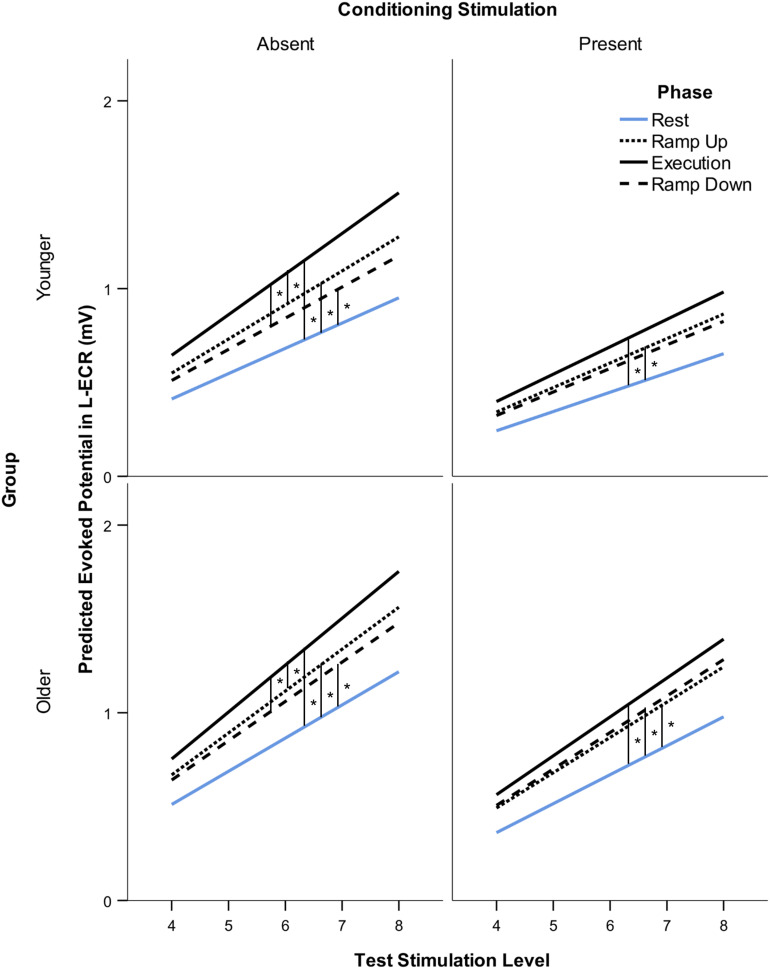
Modeled suprathreshold MEP recruitment slope in response to 5 levels of TMS stimulus intensity (from two intervals above rMT to MAX stimulation intensity) across subjects. Comparison of suprathreshold recruitment slope in the resting L-ECR across phases of R-ECR contraction for younger and older participants in the absence and presence of IHI conditioning stimulation. Asterisks indicate significant differences between slopes for each phase from the mixed-effects spline model, planned contrast *t*-test *p < 0.05.

The linear mixed-effects models of L-ECR MEP recruitment slopes were parameterized as slope-intercept models with parameters for each phase (four levels: Rest, Ramp Up, Execution, Ramp Down) and group (two levels: younger, older). In this parameterization, the slopes indicate the change in MEP in response to a change in TMS stimulus intensity (Figure 2). Because MEPs in the steep part of the recruitment (Suprathreshold) were the primary outcome of interest, we utilized a linear spline model with one knot at TMS stimulus intensity 3, corresponding to one intensity above resting motor threshold, with subject as a random effect. This model parameterization then allowed two slopes to be estimated, one across the three stimulation levels at and around resting motor threshold (Threshold MEP Recruitment Slope) and one across the five higher levels of TS (Suprathreshold MEP Recruitment Slope). This approach was confirmed during model development (described below), as parameter estimates of the Threshold slopes did not differ significantly across task phases or groups (planned contrast *t*-tests) and reducing the parameters to a single Threshold slope estimate for each CS level improved model fit (based on AIC). Thus, fitting a spline model allows us to better model the data and increase power to examine effects of interest among the Suprathreshold slopes.

Fully parameterized models were constructed separately for CS levels 0 (absent) and 3 (present). Our models included one slope and intercept parameter for each phase (Rest, Ramp Up, Execution, and Ramp Down), group (younger and older), and recruitment type (Threshold and Suprathreshold), and were estimated using maximum likelihood estimation. The data were modeled such that the intercept occurred at the rMT. These parameters were then systematically compared to reduce the model and improve model fit. Model selection was based on Akaike’s information criterion (AIC) and contrasts between parameter estimates.

Following the procedure used to analyze the L-ECR recruitment, we constructed a linear mixed-effects model of R-ECR recruitment, parameterized as slope-intercept models with parameters for each phase (four levels: Rest, Ramp Up, Execution, Ramp Down) and group (two levels: younger, older) and estimated using maximum likelihood estimation with subject as a random factor. Variables were centered such that the intercept was at the middle CS stimulation level, thus these intercepts provide a modeled estimate of the mean MEP response. These parameters were then systematically compared to reduce the model and improve model fit. Model selection was based on AIC and contrasts between parameter estimates, with the constraint that parameters of interest were retained to allow hypothesis testing and comparisons with the L-ECR model. Analogous with the L-ECR model, the covariates considered included responses in the resting L-ECR (mean MEP size and recruitment slope) and background muscle activity (EMG) in both the active and resting arms.

## Results

### Sample Characteristics and Sample Mortality

Nine individuals did not complete the study (*n* = 5 chose to withdraw their participation and *n* = 4 had technical difficulties during the TMS session that resulted in incomplete or unusable data), leaving a final total sample of *n* = 42, with *n* = 22 younger healthy participants (14 female, Range = 22–30 years old, *M* = 25.0 years, *SD* = 2.71) and *n* = 20 older healthy participants (9 female, Range = 60–80 years old, *M* = 68.1 years, *SD* = 5.25). Additional participant characteristics are given in Table 1.

### Dexterity and Strength Behavioral Results

Performance on the NHPT showed the expected differences. Younger participants were faster on the NHPT with each arm than older subjects [dominant, *t*(40) = −3.61, *p* = 0.001; non-dominant, *t*(40) = −3.96, *p* < 0.001], but the ratio of dominant/non-dominant performance did not significantly differ across groups [*t*(40) = 0.48, *p* = 0.63].

Unsurprisingly, dominant-arm MVCs were significantly stronger for men [*F*(1,38) = 9.80, *p* = 0.003] and for younger participants [*F*(1,38) = 7.99, *p* = 0.007] (Stoll et al., 2000; Decostre et al., 2015). Non-dominant arm MVCs were also stronger for men [*F*(1,38) = 12.49, *p* = 0.001], but did not differ across age groups [*F*(1,38) = 1.57, *p* = 0.22]. There were no significant interactions between sex and age in MVC measures [Dominant: *F*(1,38) = 0.87, *p* = 0.40; Non-dominant: *F*(1,38) = 0.001, *p* = 0.97].

Participants’ ratings of the force required (as a percent of their MVC) to complete the task were close to 50% and did not significantly differ across groups [*n* = 23, *t*(19) = 0.68, *p* = 0.51] (see Table 1). On average, once participants started muscle contraction, each trial lasted about 5 s, the inter-trial interval was 4 s, and trial length did not differ across groups [younger: *M* = 4726 ms, *SD* = 726, older: *M* = 4995 ms, *SD* = 829, *t*(40) = 1.12, *p* = 0.27]. To prevent fatigue participants received rest breaks every 45 trials or as needed. To evaluate fatigue objectively, R-ECR muscle activity was analyzed across EXECUTION trials. Root mean square R-ECR EMG in each EXECUTION trial was calculated for each subject over a duration of 100 ms beginning 105 ms before the TMS pulse. Correlations between trial number and root mean square EMG during Execution were near zero among all subjects (R-ECR *r* = −0.001, *p* = 0.93). There were no significant differences between mean root mean square EMG during Execution in the first third of trials compared to the last third of trials for all subjects [R-ECR *t*(41) = −0.08, *p* = 0.94; L-ECR *t*(41) = 0.79, *p* = 0.43] or among young [R-ECR *t*(21) = −0.79, *p* = 0.44] or old [R-ECR *t*(19) = 1.54, *p* = 0.14].

### Model Development

#### L-ECR Model Development

In the L-ECR linear mixed-effects model, for both CS absence and presence, there were no significant differences across groups or phases in the estimated intercept (baseline excitability at the rMT, all *p* > 0.20) or in the estimated threshold MEP recruitment slope (all *p* > 0.20). Reducing these parameters improved model fit compared to the fully parameterized model. Suprathreshold MEP recruitment showed differences among groups and phases, so these parameters were retained.

The CS absence and presence models were then combined. The fixed intercepts were significantly different (*p* < 0.001) across CS absence and presence, with an expected larger intercept (greater excitability at the rMT) for CS absence. Threshold recruitment slope parameters were also significantly greater for CS absence than CS presence (*p* < 0.001). Thus, separate intercepts and threshold slope parameters for CS absence and CS presence were retained in the model. Next, covariates that showed differences among younger and older subjects were added to the model to control for possible confounds in group differences and test for improved model fit. The covariates considered included responses in the active R-ECR (mean MEP size and recruitment slope) and background muscle activity (EMG) in both the active and resting arms. Background EMG was calculated for each subject as the root mean square of motor potential (mV) over 100 ms beginning 105 ms before any TMS pulse.

Background EMG covariates for both arms were uncorrelated with each other (*r* = 0.03, *p* = 0.24) and had significant parameter estimates, so they were retained in the final model. The other indices of R-ECR activity were not significant. Consequently, the final model consisted of 24 parameters: the variance of the random intercept and the residuals, two covariates for background EMG activity, two fixed intercepts for CS absence/presence, two threshold slopes (around the rMT) for CS absence/presence, eight suprathreshold slopes (across higher TS levels above the rMT) for each group/phase combination in the absence of CS, and eight suprathreshold slopes for each group/phase combination in the presence of high CS levels (see Table 2).

**TABLE 2:** Parameter estimates for fixed effects from the final mixed effects one-knot linear spline model of Threshold and Suprathreshold recruitment slopes for resting L-ECR.

	Group	Phase	Parameter estimate	Standard error	*t*-test (df)	*p*-value
***Fixed Intercepts***						
CS Absent	All	All	0.196	0.049	4.01 (48)	0.0002
CS Present	All	All	0.091	0.020	1.87 (47)	0.07
***Covariates***						
R EMG	—	—	0.307	0.125	2.46 (2834)	0.014
L EMG			42.1	5.44	7.73 (798)	<0.0001
***Threshold Recruitment Slope***						
CS Absent	All	All	0.122	0.020	6.01 (2813)	<0.0001
CS Present	All	All	0.034	0.013	2.69 (2813)	0.007
***Suprathreshold Recruitment Slope***						
CS Absent	Younger	Rest	0.135	0.0095	14.16 (2830)	<0.0001
		Ramp Up	0.182	0.0093	19.56 (2824)	<0.0001
		Execution	0.216	0.0095	22.73 (2826)	<0.0001
		Ramp Down	0.166	0.0093	17.88 (2828)	<0.0001
	Older	Rest	0.177	0.0099	17.92 (2837)	<0.0001
		Ramp Up	0.223	0.0097	23.01 (2827)	<0.0001
		Execution	0.250	0.0098	25.44 (2827)	<0.0001
		Ramp Down	0.209	0.0097	21.56 (2824)	<0.0001
CS Present	Younger	Rest	0.102	0.0095	10.84 (2830)	<0.0001
		Ramp Up	0.130	0.0092	14.14 (2824)	<0.0001
		Execution	0.146	0.0095	15.42 (2826)	<0.0001
		Ramp Down	0.125	0.0092	13.59 (2828)	<0.0001
	Older	Rest	0.154	0.0098	15.72 (2837)	<0.0001
		Ramp Up	0.188	0.0096	19.46 (2827)	<0.0001
		Execution	0.207	0.0098	21.21 (2827)	<0.0001
		Ramp Down	0.194	0.0096	20.12 (2824)	<0.0001

*CS, conditioning stimulus. The final model included a random intercept across subjects with a variance estimate of 0.091 (Standard Error = 0.020) and residual variance estimate of 0.138 (Standard Error = 0.004). *T*-tests indicate whether parameter estimates were significantly different from zero (two-tailed). Intercepts are expected peak-to-peak MEP amplitude (mV) at resting motor threshold. Slopes are expected change in peak-to-peak MEP amplitude for a one-level increase TS stimulation at mean background EMG levels in the resting and active arms (see sections “Statistical Analysis” and “L-ECR Model Development”).*

#### R-ECR Model Development

In the R-ECR linear mixed effects model, background EMG covariates for both arms were statistically significant and thus retained in the model. Intercepts did not significantly differ between younger and older participants (all *p* > 0.30) and thus were combined across group in the final model for R-ECR recruitment. Unlike the L-ECR model, intercepts did differ significantly across phases (see section “Contraction Phase Rather Than Contraction Force Related to Active MEP Recruitment Amplitude” below) and thus a separate intercept parameter for each phase was retained in the final R-ECR model (see Table 3).

**TABLE 3:** Parameter estimates for fixed effects from the final linear model of recruitment for active R-ECR MEPs across conditioning stimulation levels.

	Group	Phase	Parameter estimate	Standard error	*t*-test (df)	*p*-value
***Fixed Intercepts***						
		Rest	1.10	0.113	9.78 (44)	<0.0001
		Ramp Up	2.25	0.112	20.08 (42)	<0.0001
		Execution	1.88	0.113	16.66 (44)	<0.0001
		Ramp Down	1.93	0.112	17.21 (42)	<0.0001
***Covariates***						
R EMG	—	—	8.232	0.200	41.04 (4535)	<0.0001
LEMG	—	—	22.81	7.343	3.11 (3280)	0.0019
***Suprathreshold recruitment***						
TS All	Younger	Rest	0.117	0.0195	6.01 (4493)	<0.0001
		Ramp Up	0.160	0.0195	8.23 (4493)	< 0.0001
		Execution	0.180	0.0195	9.23 (4493)	<0.0001
		Ramp Down	0.234	0.0195	12.00 (4493)	<0.0001
	Older	Rest	0.133	0.0204	6.49 (4493)	<0.0001
		Ramp Up	0.145	0.0204	7.11 (4493)	<0.0001
		Execution	0.136	0.0204	6.68 (4493)	<0.0001
		Ramp Down	0.200	0.0204	9.79 (4493)	<0.0001

*TS, test stimulus. The final model included a random intercept across subjects with a variance estimate of 0.515 (Standard Error = 0.114) and residual variance estimate of 0.339 (Standard Error = 0.007). *T*-tests indicate whether parameter estimates were significantly different from zero (two-tailed). Intercepts are expected peak-to-peak MEP amplitude (mV) at the middle CS stimulation level at mean background EMG levels in the resting and active arms. Slopes are expected change in peak-to-peak MEP amplitude for a one-level increase in equivalent TS-stimulation units at mean background EMG levels in the resting and active arms (see sections “Statistical Analysis” and “R-ECR Model Development”). Model fit parameters: AIC = 8208, −2 RLL = 8176.*

### Resting L-ECR MEP Recruitment Slope During R-ECR Contraction

#### Contraction Force Rather Than Contraction Phase Related to Resting MEP Recruitment Slope

Suprathreshold MEP recruitment slopes in the L-ECR during R-ECR Ramp Up, Execution, and Ramp Down were significantly higher compared to Rest, for both younger and older subjects (see Table 4 and Figure 2). Interestingly, L-ECR slopes during R-ECR Ramp Up and Ramp Down did not differ from each other in either group, despite the phase of contraction being opposite in the two conditions (younger: slope difference = 0.016, *p* = 0.19; older: slope difference = 0.014, *p* = 0.27). L-ECR slopes during Execution were significantly greater than slopes during Ramp Up (younger: slope difference = 0.035, *p* = 0.003; older: slope difference = 0.027, *p* = 0.032) and Ramp Down (younger: slope difference = 0.050, *p* < 0.001; older: slope difference = 0.040, *p* = 0.001).

**TABLE 4:** Difference in suprathreshold recruitment slope between active phases and rest in the presence and absence of conditioning stimulation (CS) for the resting L-ECR, controlling for active R-ECR EMG and MEP.

CS	Group	Ramp Up – Rest	Execution – Rest	Ramp Down – Rest
Absent	Younger	0.047***	0.082***	0.031**
	Older	0.046***	0.073***	0.033**
	Older – Younger	−0.0003	−0.009	0.001
Present	Younger	0.028*	0.043***	0.023
	Older	0.034**	0.053***	0.040**
	Older – Younger	0.006	0.010	0.017
Present – Absent	Younger	−0.019	−0.038*	−0.008
	Older	−0.013	−0.020	0.007

Asterisks indicate slope difference between the active phase and rest that were significantly different from zero from the mixed-effects spline model, planned contrast *t*-test*p < 0.05,**p < 0.01,***p < 0.001 (two-tailed).

#### Age and IHI

**FIGURE 3:**
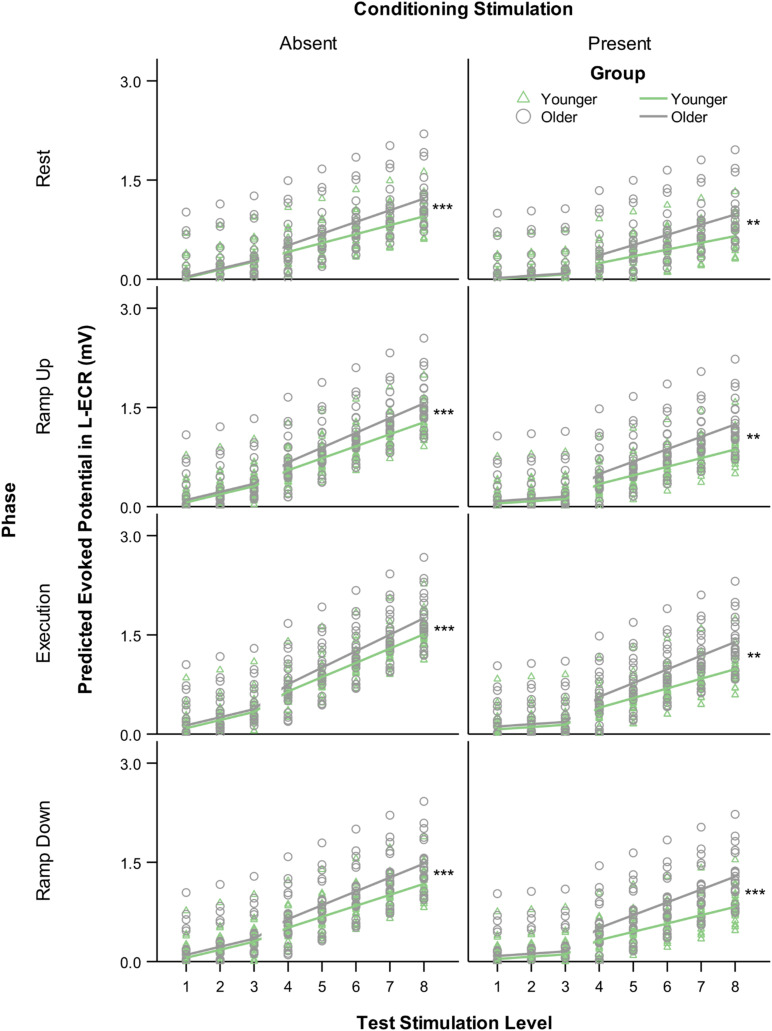
Modeled MEP recruitment slopes in the L-ECR for each phase of isometric wrist extensor contraction as a function of TS level comparing younger versus older participants in the absence **(left)** and presence **(right)** of conditioning stimulation (CS). Values were generated from a linear mixed-effects spline model with one knot at TS level 3. Asterisks indicate significant differences between younger and older groups in suprathreshold slopes from the mixed-effects model, planned contrast *t*-test **p < 0.01, ***p < 0.001.

The most significant finding regarding the effects of age was that for all phases of R-ECR isometric wrist extensor contraction, older participants had higher resting L-ECR MEP recruitment slopes than younger participants, in both the absence and presence of CS (see Table 4 and Figure 3). As expected, the CS did have a significant inhibitory effect on the Threshold MEP Recruitment Slope across groups and phases [−0.088, *t*(2813) = 3.69 *p* < 0.001]. For Suprathreshold MEP Recruitment Slopes, CS presence had a more complicated effect. For younger subjects, CS significantly inhibited Suprathreshold MEP Recruitment Slopes across all phases (Rest: −0.032, *p* = 0.012, Ramp Up: −0.051, *p* < 0.001, Execution: −0.070, *p* < 0.001, Ramp Down: −0.040, *p* = 0.001). In younger subjects, this interhemispheric inhibitory effect reduced the typically excitatory effect of the Execution task on the MEP Recruitment Slope compared to Rest (−0.038, *p* = 0.022). For older subjects, CS lowered MEP Recruitment Slopes significantly only in Ramp Up and Execution (Rest: −0.022, *p* = 0.091, Ramp Up: −0.035, *p* = 0.008, Execution: −0.043, *p* = 0.001, Ramp Down: −0.015, *p* = 0.26) and had no statistically significant effect on contraction-induced changes in MEP Recruitment Slope (see Table 5 and Figure 2).

**TABLE 5:** Differences in Suprathreshold MEP Recruitment Slope between older and younger subjects and between presence and absence of conditioning stimulation (CS) across phases of isometric wrist extensor contraction for the resting L-ECR, controlling for active R-ECR EMG and MEP.

CS	Group	Rest	Ramp Up	Execution	Ramp Down
Absent	Older – Younger	0.042**	0.042**	0.033**	0.043***
Present	Older – Younger	0.052***	0.057***	0.061***	0.069***
Present – Absent	Younger	−0.032*	−0.051***	−0.070***	−0.040**
Present – Absent	Older	−0.022	−0.035**	−0.043**	−0.015

Positive numbers indicate steeper slopes for older participants. Asterisks indicate group differences significantly different from zero from the mixed-effects spline model, planned contrast *t*-test *p < 0.05, **p < 0.01, ***p < 0.001 (two-tailed).

#### Comparison to IHI as a Percentage

**FIGURE 4:**
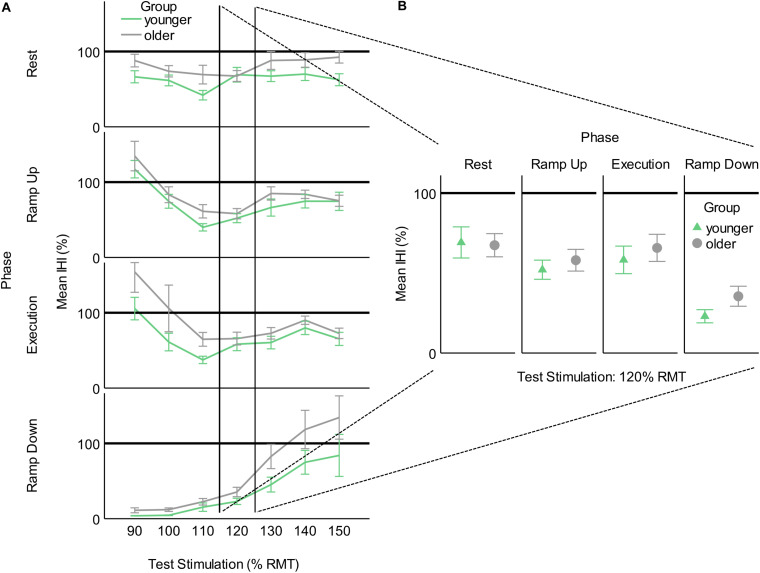
Interhemispheric inhibition calculated as a percentage of conditioned MEP to unconditioned MEP for younger and older participants across task phases. **(A)** As a function of test stimulation (percent of resting motor threshold [rMT]). **(B)** At the traditional test stimulation intensity of 120% rMT. This figure illustrates the added information provided by utilizing MEP recruitment slopes gathered across multiple stimulation intensities. Values below 100% indicate inhibition and values above 100% indicate facilitation.

By obtaining MEP recruitment slopes at rest and then measuring the effects of behavior and inhibition on those MEP recruitment slopes, while controlling for opposite limb MEP recruitment slopes, we more robustly differentiated the effects of behavior and IHI. However, for comparison with previous studies, we also calculated test stimulation intensities in percentage of rMT for each subject and binned these in 10% increments. This procedure yielded a useful range of TMS test stimulation intensities of 90–150% rMT. We then calculated IHI as a percentage by dividing the average conditioned MEP by the unconditioned MEP and multiplying by 100 for each subject and plotting these percentages for each group in each task phase (Figure 4). The challenge with studying IHI only as a percentage is demonstrated when we examine the values across a range of TS intensities (Figure 4A). The amount of inhibition reported when utilizing a percentage varies non-linearly depending on the level of recruitment of the receiving corticospinal system. Furthermore, it is evident that testing IHI at only 1 or a few TS stimulation intensities would have missed old/young differences that we detected by modeling MEP recruitment slopes using multiple TS intensities (Figure 4B).

### Active R-ECR MEP Recruitment

#### Contraction Phase Rather Than Contraction Force Related to Active MEP Recruitment Slope

**FIGURE 5:**
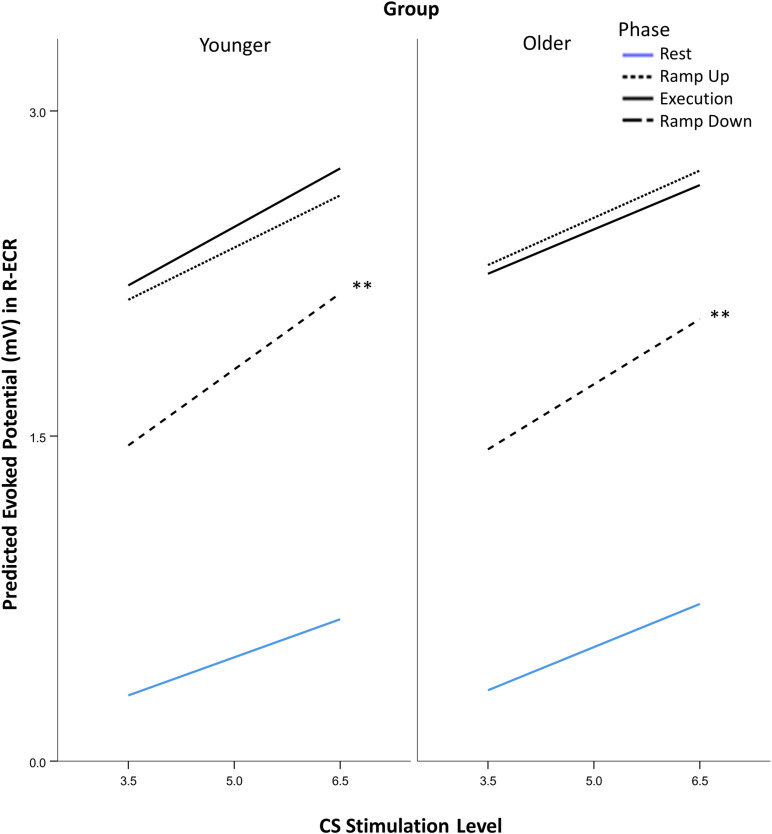
Comparison of suprathreshold corticospinal recruitment in the active R-ECR across phases of contraction for younger and older participants across conditioning stimulation (CS). See section “Statistical Analysis” for explanation of stimulation levels graphed on the *x*-axis. Asterisks indicate that Ramp Down slopes were significant different from each of the other phases from the mixed-effects liner model, planned contrast *t*-test **p < 0.01.

Recruitment slopes in the active R-ECR did not differ significantly across younger and older participants for any of the task phases (all *p* > 0.12; see Figure 5). Across both groups, recruitment slopes during Ramp Down were significantly steeper compared to any of the other phases (Rest: 0.184, *p* < 0.001; Ramp Up: 0.128, *p* = 0.001; Execution: 0.118, *p* = 0.003). There were no significant differences between slopes among the other phases (Ramp up vs. Rest: 0.056, *p* = 0.61; Execution vs. Rest: 0.066, *p* = 0.10; Ramp up vs. Execution: 0.044, *p* = 0.27).

#### Contraction Phase Rather Than Contraction Force Related to Active MEP Recruitment Amplitude

Mean peak-to-peak MEP amplitudes, at the middle CS stimulation level in the linear mixed model, in the active R-ECR were not significantly different between younger and older participants (all *p* > 0.30) but there were significant differences across task phases. All active phases had greater active mean MEP amplitudes compared to Rest (all *p* < 0.001). Unadjusted active mean MEPs during Ramp Down were significantly lower compared to Ramp Up (−0.660, *p* < 0.001) and Execution (−0.684, *p* < 0.001), whereas Ramp Up and Execution did not significantly differ from one another (0.024, *p* = 0.40).

**FIGURE 6:**
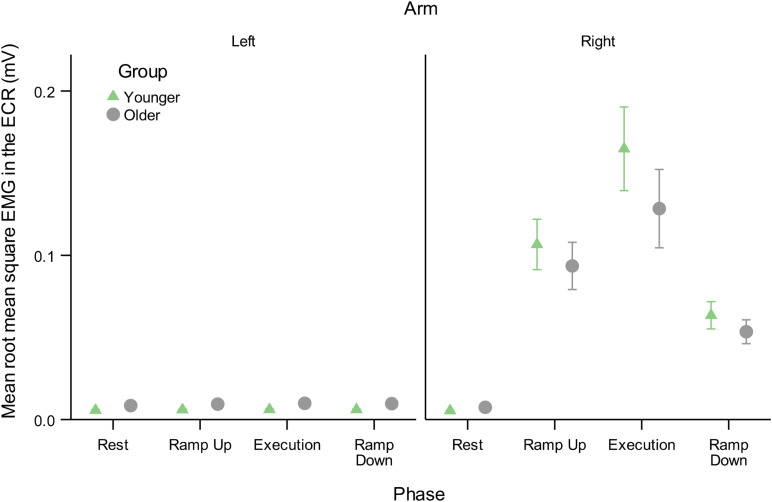
Mean root mean square of EMG background muscle activity in the resting L-ECR and active R-ECR across phases for younger and older participants. Error bars are ±1 SEM. For the R-ECR, all active phases of contraction were significantly different from each other and from rest (planned contrast *t*-tests, all *p* < 0.05, see section “Contraction Phase Rather Than Contraction Force Related to Active MEP Recruitment Amplitude”). There were no significant differences among any phases in the L-ECR [*F*(3,126) = 1.03, *p* = 0.38; see Table 6].

However, a mixed-effects model including AGE and PHASE parameters showed mean background EMG in the active R-ECR differed across all phases [*F*(3,126) = 62.21, *p* < 0.001], with the greatest activity for Execution, as expected (see Table 6 and Figure 6). All active phases of contraction had significantly greater EMG activity compared to Rest (Ramp Up:0.094, *p* < 0.001; Execution:0.142, *p* < 0.001; Ramp Down:0.053, *p* = 0.004). Background EMG was greater in Execution compared to both Ramp Up (0.048, *p* = 0.03) and Ramp Down (0.089, *p* < 0.001), and Ramp Up activity was significantly greater than Ramp Down (0.041, *p* = 0.01). Mean background R-ECR EMG did not significantly differ across older and younger subjects (−0.002, *p* = 0.37), nor was there an interaction between AGE and PHASE with regards to background R-ECR EMG [*F*(3,126) = 1.13, *p* = 0.34].

**TABLE 6:** Mean background EMG levels (mV) in the L-ECR and R-ECR across all stimulation levels.

Variable	Group	Rest	Ramp Up	Execution	Ramp Down
Mean L-ECR EMG (Standard Deviation)	Young	0.006 (0.002)	0.006 (0.002)	0.006 (0.002)	0.006 (0.002)
	Old**	0.009 (0.008)	0.010 (0.006)	0.010 (0.006)	0.010 (0.006)
	All	0.007 (0.007)	0.008 (0.005)	0.008 (0.005)	0.008 (0.005)
Mean R-ECR EMG (Standard Deviation)	Young	0.005 (0.001)	0.106*** (0.072)	0.165*** (0.119)	0.063*** (0.039)
	Old	0.007 (0.003)	0.094*** (0.064)	0.128*** (0.106)	0.053*** (0.032)
	All	0.006 (0.003)	0.100*** (0.070)	0.148*** (0.115)	0.059** (0.037)

Asterisks indicate significant mean differences in a mixed-effects model, planned contrast *t*-test, **p < 0.01, ***p < 0.001 (two-tailed). L-ECR: group differences between young and old. R-ECR: active phases different from rest.

Thus, background EMG was a confounding factor necessary to evaluate the effects of contraction phase on Active arm MEP amplitudes. After controlling for background EMG, active mean MEP amplitudes during Ramp Up were significantly greater compared to Ramp Down (0.322, *p* < 0.001), despite force production being identical (25% MVC) in the two conditions. R-ECR MEP amplitudes during Ramp Up were also significantly greater compared to Execution (0.371, *p* < 0.001), despite the force production being lower (25% vs. 50% MVC). Execution and Ramp Down did not differ significantly (0.049, *p* = 0.10). Thus, in contrast to the resting L-ECR, R-ECR MEP amplitudes during Execution were related to greater preceding background EMG activity.

## Discussion

### Summary of Results

The novel contribution of this study was the demonstration that contraction force and contraction phase correlate with different changes in MEP recruitment slope such that: (1) in the resting arm, MEP recruitment slope was dependent on the force level produced by the opposite arm irrespective of the phase of muscle contraction, and controlled for active arm MEP recruitment slope (see section “Contraction Force Rather Than Contraction Phase Related to Resting MEP Recruitment Slope”); (2) in the active arm, MEP recruitment amplitude was dependent on the phase of muscle contraction irrespective of force production (see section “Contraction Phase Rather Than Contraction Force Related to Active MEP Recruitment Amplitude”); and (3) in older age, resting arm MEP recruitment slope was higher at rest and throughout contralateral contraction (see section “Contraction Force Rather Than Contraction Phase Related to Resting MEP Recruitment Slope”) and IHI was reduced in a phase dependent manner compared to younger (see section “Age and IHI”).

### Force-Dependent and Phase-Dependent MEP Recruitment Slope Effects

The unique aspect of Ramp Up and Ramp Down phases was that the R-ECR was producing the same amount of force in each condition (25% of MVC); however, the force change was in the opposite direction (i.e., increasing in Ramp Up and decreasing in Ramp Down). This design allowed us to look for a potential dissociation between Contraction Force and Contraction Phase, the latter mirroring the state of nervous system drive. Our results indicate that MEP recruitment slope in the resting limb correlates with the level of force being produced by the isometrically contracting opposite limb, rather than the direction of force change. This was demonstrated in the equally increased MEP recruitment slope in the L-ECR at both Ramp Up and Ramp Down compared to Rest (Table 4; younger: slope difference = 0.016, *p* = 0.19; older: slope difference = 0.014, *p* = 0.27). Coupling and uncoupling of contralateral homologous limbs occurs through changes in functional activation and inhibition interhemispherically (Boisgontier et al., 2014). Our results further expand on the understanding of interlimb coupling by demonstrating that MEP recruitment slope in the homologous muscle is increased, even when it remains at rest, and mirrors the force being produced by the opposite limb.

The force-dependent MEP recruitment slope in the resting L-ECR contrasted with what we found in the active R-ECR. Instead, we found that R-ECR recruitment slope, controlled for background EMG, was significantly steeper only in Ramp Down compared to all other phases (Rest: 0.184, *p* < 0.001; Ramp Up: 0.128, *p* = 0.001; Execution: 0.118, *p* = 0.003). And R-ECR recruitment slope did not differ between young and old (*p* > 0.12). Similarly to what is observed in fatigued muscles via the method of twitch interpolation (Gandevia, 2001), this finding likely demonstrates that during isometric relaxation, there is reduced central drive. On the other hand, R-ECR MEP amplitudes in the active ECR seem to reflect the state of nervous system drive, as represented by Contraction Phase or direction of force change. R-ECR MEP recruitment amplitude was greater during Ramp Up than Ramp Down despite the same level of force being produced (−0.660, *p* < 0.001). This finding is consistent with the idea that motor cortical activity is believed to contribute to muscle relaxation (Terada et al., 1995). The degree of contribution is likely influenced by the type of contraction and the speed and type of relaxation (Rothwell et al., 1998).

### Impact of Aging on MEP Recruitment Slope and IHI

The predominant finding in prior work is that of reduced intracortical inhibition, increased MEP recruitment slope, and predominantly decreased transcallosal inhibition in healthy aging (Peinemann et al., 2001; Silbert et al., 2006; Fling and Seidler, 2011, 2012; Levin et al., 2011, 2014; Heise et al., 2013; Petitjean and Ko, 2013; Plow et al., 2013; Coppi et al., 2014; Papegaaij et al., 2014; Opie et al., 2015). Less frequently, excessive intracortical inhibition and reduced excitability have been found with age. The direction of findings may, in fact, be dependent on the specific phenomena being studied and the impairments present in the particular population examined (Clark and Taylor, 2011; Clark et al., 2015). Our findings of increased MEP recruitment slope throughout the phases of contralateral muscle contraction (Table 4 and Figure 3) and reduced short interval IHI in two of four phases of contralateral muscle contraction (Ramp Up: −0.035, *p* = 0.008, Execution: −0.043, *p* = 0.001) are consistent with the majority of the previous literature. Our investigation further adds to previous studies by demonstrating that the age-related reduction in short interval IHI was absent during Ramp Up and Execution, where we found the presence of a transcallosal effect on MEP recruitment slope even in older participants. The phase-dependent nature of these transcallosal effects may implicate one mechanism whereby exercise or repetitive movements have been demonstrated to enhance cortical inhibition (Mierau et al., 2014). The majority of the cited literature found age-related increases in MEP amplitudes, and decreases in intracortical paired pulse inhibition, ipsilateral silent period, or long interval IHI. In the studies that have specifically examined short interval IHI, Talelli et al. (2008a,b) only found an age effect in long interval IHI. Plow et al. (2013) found greater levels of paired-pulse short interval IHI at rest in their older subjects. This finding is not surprising nor contrary to what we found when testing and comparing IHI at one stimulus level between young and old (see Figure 4). It is for this reason that we used our method of comparing MEP recruitment slopes and the effects of contralateral conditioning pulses on the MEP recruitment slopes, rather than testing IHI at only one stimulation intensity.

### Modulation and Measurement of IHI

Since the first description of non-invasively recorded IHI in humans, it has been understood that voluntary activity in the conditioning corticospinal system increases the reduction in MEP amplitude induced by contralateral conditioning stimuli (Ferbert et al., 1992). The greater challenge has been differentiating the effects of voluntary activity on the sending corticospinal system versus its effects on the receiving corticospinal system. Several methods have attempted to control for the unmeasurable interaction between sending and receiving corticospinal systems, including changing stimulus intensities in order to match amplitude values in the denominator of the percentage measurement. Changing stimulus intensities presumes that stimulus intensities affect corticospinal and cortico-cortical circuits identically. In actuality, there is recognition that IHI does not have a uniform effect on corticospinal recruitment as a whole, since IHI can reduce short-interval-intracortical-inhibition (SICI) and potentially contribute to overall increases in corticospinal recruitment (Daskalakis et al., 2002; Perez and Cohen, 2009; Perez et al., 2014). To reduce our dependence on the assumptions inherent in matching MEP amplitudes via stimulus intensity manipulations, we instead obtained IHI recruitment across levels of Test Stimulus intensities and modeled the slopes controlling for covariates, a technique previously demonstrated to enhance reliability (Butefisch et al., 2008; Heise et al., 2013). Through this method we were able to demonstrate in young healthy participants the presence of sending IHI at all phases of isometric contraction (Rest: −0.032, *p* = 0.012, Ramp Up: −0.051, *p* < 0.001, Execution: −0.070, *p* < 0.001, Ramp Down: −0.040, *p* = 0.001) and a significant increase in IHI effects on MEP recruitment slope only at execution (−0.038, *p* = 0.022) and not ramp-up and ramp-down (*p*’s > 0.05). In older healthy participants, we found a lack of IHI effect at rest (−0.022, *p* = 0.091) and ramp-down (−0.015, *p* = 0.26), but a presence of IHI effects during ramp-up (−0.035, *p* = 0.008) and execution (−0.043, *p* = 0.001; see Table 5 and Figure 2). This expands on previous literature by demonstrating that healthy aging effects on interhemispheric interactions are task-specific and that remnants of overall reduced IHI effects in older age are able to be activated under certain conditions.

### Limitations and Future Opportunities

Our study was limited in the following ways: We investigated only unilateral isometric muscle contractions of the dominant limb and their effects on the non-dominant limb. We also only tested IHI from the active to resting M1. These were both practical limitations necessary given the large amount of time dedicated to obtaining the parametrized recruitment samples in each hemisphere. Testing the limbs in opposite configurations may be important as some studies have found a reduction in hemispheric lateralization in hand dominance with age, though others have not (Bernard and Seidler, 2012; Sebastjan et al., 2017), and IHI from the rest to active M1 is clearly important for release of inhibition required for activity (Murase et al., 2004). This study was also not adequately powered to evaluate gender differences. Our technique of determining our recruitment stimulation intensity range, by capping the maximal intensity and sampling at equal intervals across the range, as opposed to the traditional method of starting at resting motor threshold and sampling at defined percentages of that intensity, is novel. In its novelty, our technique did adequately achieve our goals, which was to sample at the linear portion of the corticospinal recruitment curve in an efficient manner and reduce variance in comparing participants with very different resting motor thresholds. However, our novel method is limited in that if there is a non-linear effect on the plateau portion of corticospinal recruitment, we would miss it. It is also limited in that it may make it difficult to compare our results to other studies that either use the traditional method of sampling or only sample at one or two intensity levels. Additionally, our study was limited in only studying one form of IHI, specifically short-interval IHI. Both long-interval IHI (Fling et al., 2011) and ipsilateral silent period (Giovannelli et al., 2009) have been demonstrated to be important circuits that are modulated by muscle contraction and altered in aging. Whether those circuits show similar changes related to force production and phase of contraction has yet to be determined.

While we monitored other arm muscles, both to set the neuroanatomically relevant stimulation intensity range for our MEP recruitment and to monitor task performance, we did not design the study to measure corticospinal activity of our non-target muscles. Future studies should investigate the somatotopic specificity of corticospinal and interhemispheric interactions as they are clearly important for training and transfer (Ruddy et al., 2017; Carson and Rankin, 2018; Chye et al., 2018). With the framework we have designed, future studies can interrogate multiple forms of unimanual and bimanual activity, and delve further into differences in force production, MEP recruitment, other circuits, and the influence of aging. As demonstrated elsewhere, current modalities for enhancing motor control and recovery after nervous system injury are often monotonic and applied either before or continuously throughout a behavioral task. Our results contribute to the foundational understanding of MEP recruitment in relation to behavior and will aid in the design of dynamically specific interventions to more effectively enhance function.

## Data Availability Statement

The raw data supporting the conclusions of this article will be made available by the authors, without undue reservation.

## Ethics Statement

The studies involving human participants were reviewed and approved by Institutional Review Board (IRB) of the University of Maryland, Baltimore. The patients/participants provided their written informed consent to participate in this study.

## Author Contributions

MD and EE: concept and design. MD, EE, JL, LM, JW, and GW: acquisition, analysis, and interpretation. MD, EE, SH, LM, JW, and GW: drafting and revising. All authors contributed to the article and approved the submitted version.

## Conflict of Interest

The authors declare that the research was conducted in the absence of any commercial or financial relationships that could be construed as a potential conflict of interest.

## References

[B1] Addamo P. K. Farrow M. Hoy K. E. Bradshaw J. L. Georgiou-Karistianis N. (2007). The effects of age and attention on motor overflow production–A review. *Brain Res. Rev.* 54 189–204. 10.1016/j.brainresrev.2007.01.004 17300842

[B2] Bernard J. A. Seidler R. D. (2012). Hand dominance and age have interactive effects on motor cortical representations. *PLoS One* 7:e45443. 10.1371/journal.pone.0045443 23049800 PMC3458089

[B3] Boddington L. J. Reynolds J. N. (2017). Targeting interhemispheric inhibition with neuromodulation to enhance stroke rehabilitation. *Brain Stimul.* 10 214–222. 10.1016/j.brs.2017.01.006 28117178

[B4] Boisgontier M. P. Wittenberg G. F. Fujiyama H. Levin O. Swinnen S. P. (2014). Complexity of central processing in simple and choice multilimb reaction-time tasks. *PLoS One* 9:e90457. 10.1371/journal.pone.0090457 24587371 PMC3938735

[B5] Butefisch C. M. Wessling M. Netz J. Seitz R. J. Homberg V. (2008). Relationship between interhemispheric inhibition and motor cortex excitability in subacute stroke patients. *Neurorehabil. Neural Rep.* 22 4–21. 10.1177/1545968307301769 17507644

[B6] Carson R. G. Rankin M. L. (2018). Shaping the effects of associative brain stimulation by contractions of the opposite limb. *Front. Psychol.* 9:2249. 10.3389/fpsyg.2018.02249 30510533 PMC6252341

[B7] Chen R. Yung D. Li J. Y. (2003). Organization of ipsilateral excitatory and inhibitory pathways in the human motor cortex. *J. Neurophysiol.* 89 1256–1264. 10.1152/jn.00950.200200950.2002 12611955

[B8] Chye L. Riek S. de Rugy A. Carson R. G. Carroll T. J. (2018). Unilateral movement preparation causes task-specific modulation of TMS responses in the passive, opposite limb. *J. Physiol.* 596 3725–3738. 10.1113/JP275433 29775218 PMC6092291

[B9] Cincotta M. Ziemann U. (2008). Neurophysiology of unimanual motor control and mirror movements. *Clin. Neurophysiol.* 119 744–762. 10.1016/j.clinph.2007.11.047 18187362

[B10] Clark B. C. Taylor J. L. (2011). Age-related changes in motor cortical properties and voluntary activation of skeletal muscle. *Curr. Aging Sci.* 4:192. 10.2174/1874609811104030192 21529329 PMC3184350

[B11] Clark B. C. Taylor J. L. Hong S. L. Law T. D. Russ D. W. (2015). Weaker Seniors Exhibit motor cortex hypoexcitability and impairments in voluntary activation. *J. Gerontol. A Biol. Sci. Med. Sci* 70 1112–1119. 10.1093/gerona/glv030 25834195 PMC4861648

[B12] Coppi E. Houdayer E. Chieffo R. Spagnolo F. Inuggi A. Straffi L. (2014). Age-related changes in motor cortical representation and interhemispheric interactions: a transcranial magnetic stimulation study. *Front. Aging Neurosci.* 6:209. 10.3389/fnagi.2014.00209 25157232 PMC4128298

[B13] Cram J. R. Kasman G. S. Holtz J. (1998). *Introduction to Surface Electromyography.* Gaithersburg, MD: Aspen Publishers, Inc.

[B14] Cuypers K. Thijs H. Meesen R. L. (2014). Optimization of the transcranial magnetic stimulation protocol by defining a reliable estimate for corticospinal excitability. *PLoS One* 9:e86380. 10.1371/journal.pone.0086380 24475111 PMC3901672

[B15] Daskalakis Z. J. Christensen B. K. Fitzgerald P. B. Roshan L. Chen R. (2002). The mechanisms of interhemispheric inhibition in the human motor cortex. *J. Physiol.* 543(Pt 1), 317–326. 10.1113/jphysiol.2002.017673 12181302 PMC2290496

[B16] Decostre V. Canal A. Ollivier G. Ledoux I. Moraux A. Doppler V. (2015). Wrist flexion and extension torques measured by highly sensitive dynamometer in healthy subjects from 5 to 80 years. *BMC Musculoskelet. Disord.* 16:4. 10.1186/s12891-015-0458-9 25636264 PMC4322806

[B17] Di Lazzaro V. Oliviero A. Profice P. Insola A. Mazzone P. Tonali P. (1999). Direct demonstration of interhemispheric inhibition of the human motor cortex produced by transcranial magnetic stimulation. *Exp. Brain Res*. 124, 520–524. 10090664 10.1007/s002210050648

[B18] Dimyan M. A. Perez M. A. Auh S. Tarula E. Wilson M. Cohen L. G. (2014). Nonparetic arm force does not overinhibit the paretic arm in chronic poststroke hemiparesis. *Arch. Phys. Med. Rehabil.* 95 849–856. 10.1016/j.apmr.2013.12.023 24440364 PMC4004647

[B19] Ferbert A. Priori A. Rothwell J. C. Day B. L. Colebatch J. G. Marsden C. D. (1992). Interhemispheric inhibition of the human motor cortex. *J. Physiol.* 453 525–546. 10.1113/jphysiol.1992.sp019243 1464843 PMC1175572

[B20] Fling B. W. Peltier S. J. Bo J. Welsh R. C. Seidler R. D. (2011). Age differences in interhemispheric interactions: callosal structure, physiological function, and behavior. *Front. Neurosci.* 5:38. 10.3389/fnins.2011.00038 21519384 PMC3077973

[B21] Fling B. W. Seidler R. D. (2011). Fundamental differences in callosal structure, neurophysiologic function, and bimanual control in young and older adults. *Cereb. Cortex* 22 2643–2652. 10.1093/cercor/bhr349 22166764 PMC3464417

[B22] Fling B. W. Seidler R. D. (2012). Task-dependent effects of interhemispheric inhibition on motor control. *Behav. Brain Res.* 226 211–217. 10.1016/j.bbr.2011.09.018 21944939 PMC3208314

[B23] Fujiyama H. Garry M. I. Levin O. Swinnen S. P. Summers J. J. (2009). Age-related differences in inhibitory processes during interlimb coordination. *Brain Res* 1262 38–47. 10.1016/j.brainres.2009.01.023 19368842

[B24] Fujiyama H. Van Soom J. Rens G. Gooijers J. Leunissen I. Levin O. (2016). Age-related changes in frontal network structural and functional connectivity in relation to bimanual movement control. *J. Neurosci.* 36 1808–1822. 10.1523/JNEUROSCI.3355-15.2016 26865607 PMC6602021

[B25] Gandevia S. C. (2001). Spinal and supraspinal factors in human muscle fatigue. *Physiol. Rev.* 81 1725–1789. 10.1152/physrev.2001.81.4.1725 11581501

[B26] Giovannelli F. Borgheresi A. Balestrieri F. Zaccara G. Viggiano M. P. Cincotta M. (2009). Modulation of interhemispheric inhibition by volitional motor activity: an ipsilateral silent period study. *J. Physiol.* 587(Pt. 22), 5393–5410. 10.1113/jphysiol.2009.175885 19770195 PMC2793872

[B27] Gueorguieva R. Krystal J. H. (2004). Move over ANOVA: progress in analyzing repeated-measures data and its reflection in papers published in the archives of general psychiatry. *Arch. Gen. Psychiatry* 61 310–317. 10.1001/archpsyc.61.3.310 14993119

[B28] Heise K. F. Zimerman M. Hoppe J. Gerloff C. Wegscheider K. Hummel F. C. (2013). The aging motor system as a model for plastic changes of GABA-mediated intracortical inhibition and their behavioral relevance. *J. Neurosci.* 33 9039–9049. 10.1523/JNEUROSCI.4094-12.2013 23699515 PMC6705012

[B29] Hidler J. Hodics T. Xu B. Dobkin B. Cohen L. G. (2006). MR compatible force sensing system for real-time monitoring of wrist moments during fMRI testing. *J. Neurosci. Methods* 155 300–307. 10.1016/j.jneumeth.2006.01.016 16490258 PMC4162675

[B30] Hinder M. R. (2012). Interhemispheric connectivity between distinct motor regions as a window into bimanual coordination. *J. Neurophysiol.* 107 1791–1794. 10.1152/jn.00822.2011 22131373

[B31] Hinder M. R. Schmidt M. W. Garry M. I. Summers J. J. (2010). Unilateral contractions modulate interhemispheric inhibition most strongly and most adaptively in the homologous muscle of the contralateral limb. *Exp. Brain Res.* 205 423–433. 10.1007/s00221-010-2379-z 20686888

[B32] Kimura T. Yamanaka K. Nozaki D. Nakazawa K. Miyoshi T. Akai M. (2003). Hysteresis in corticospinal excitability during gradual muscle contraction and relaxation in humans. *Exp. Brain Res.* 152 123–132. 10.1007/s00221-003-1518-1 12879181

[B33] Koerte I. Eftimov L. Laubender R. P. Esslinger O. Schroeder A. S. Ertl-Wagner B. (2010). Mirror movements in healthy humans across the lifespan: effects of development and ageing. *Dev. Med. Child Neurol.* 52 1106–1112. 10.1111/j.1469-8749.2010.03766.x 21039436

[B34] Kukke S. N. Paine R. W. Chao C. C. de Campos A. C. Hallett M. (2014). Efficient and reliable characterization of the corticospinal system using transcranial magnetic stimulation. *J. Clin. Neurophysiol.* 31 246–252. 10.1097/WNP.0000000000000057 24887609 PMC4744647

[B35] Levin O. Cuypers K. Netz Y. Thijs H. Nuttin B. Helsen W. F. (2011). Age-related differences in human corticospinal excitability during simple reaction time. *Neurosci. Lett.* 487 53–57. 10.1016/j.neulet.2010.09.072 20932881

[B36] Levin O. Fujiyama H. Boisgontier M. P. Swinnen S. P. Summers J. J. (2014). Aging and motor inhibition: a converging perspective provided by brain stimulation and imaging approaches. *Neurosci. Biobehav. Rev.* 43 100–117. 10.1016/j.neubiorev.2014.04.001 24726575

[B37] Liuzzi G. Horniss V. Lechner P. Hoppe J. Heise K. Zimerman M. (2013). Development of movement-related intracortical inhibition in acute to chronic subcortical stroke. *Neurology* 82 198–205. 10.1212/wnl.0000000000000028 24353337

[B38] Mathiowetz V. Weber K. Kashman N. Volland G. (2016). Adult norms for the nine hole peg test of finger dexterity. *Occupat. Ther. J. Res.* 5 24–38. 10.1177/153944928500500102

[B39] McGinley M. Hoffman R. L. Russ D. W. Thomas J. S. Clark B. C. (2010). Older adults exhibit more intracortical inhibition and less intracortical facilitation than young adults. *Exp. Gerontol.* 45 671–678. 10.1016/J.Exger.2010.04.005 20417265 PMC2926152

[B40] Meier J. D. Aflalo T. N. Kastner S. Graziano M. S. (2008). Complex organization of human primary motor cortex: a high-resolution fMRI study. *J. Neurophysiol.* 100 1800–1812. 10.1152/jn.90531.2008 18684903 PMC2576195

[B41] Mierau A. Hulsdunker T. Mierau J. Hense A. Hense J. Struder H. K. (2014). Acute exercise induces cortical inhibition and reduces arousal in response to visual stimulation in young children. *Int. J. Dev. Neurosci.* 34 1–8. 10.1016/j.ijdevneu.2013.12.009 24412583

[B42] Moller C. Arai N. Lucke J. Ziemann U. (2009). Hysteresis effects on the input-output curve of motor evoked potentials. *Clin. Neurophysiol.* 120 1003–1008. 10.1016/j.clinph.2009.03.001 19329358

[B43] Murase N. Duque J. Mazzocchio R. Cohen L. G. (2004). Influence of interhemispheric interactions on motor function in chronic stroke. *Ann. Neurol.* 55 400–409. 10.1002/ana.10848 14991818

[B44] Nasreddine Z. S. Phillips N. Chertkow H. (2012). Normative data for the Montreal Cognitive Assessment (MoCA) in a population-based sample. *Neurology* 78 765–766. 10.1212/01.wnl.0000413072.54070.a3 22391608

[B45] Nasreddine Z. S. Phillips N. A. Bedirian V. Charbonneau S. Whitehead V. Collin I. (2005). The montreal cognitive assessment, MoCA: a brief screening tool for mild cognitive impairment. *J. Am. Geriatr. Soc.* 53 695–699. 10.1111/j.1532-5415.2005.53221.x 15817019

[B46] Oldfield R. C. (1971). The assessment and analysis of handedness: the Edinburgh inventory. *Neuropsychologia* 9 97–113. 10.1016/0028-3932(71)90067-4 5146491

[B47] Opie G. M. Ridding M. C. Semmler J. G. (2015). Age-related differences in pre- and post-synaptic motor cortex inhibition are task dependent. *Brain Stimul.* 8 926–936. 10.1016/j.brs.2015.04.001 25944419

[B48] Papegaaij S. Taube W. Hogenhout M. Baudry S. Hortobagyi T. (2014). Age-related decrease in motor cortical inhibition during standing under different sensory conditions. *Front. Aging Neurosci.* 6:126. 10.3389/fnagi.2014.00126 24971063 PMC4054792

[B49] Peinemann A. Lehner C. Conrad B. Siebner H. R. (2001). Age-related decrease in paired-pulse intracortical inhibition in the human primary motor cortex. *Neurosci. Lett.* 313 33–36. 10.1016/s0304-3940(01)02239-x 11684333

[B50] Perez M. A. Butler J. E. Taylor J. L. (2014). Modulation of transcallosal inhibition by bilateral activation of agonist and antagonist proximal arm muscles. *J. Neurophysiol.* 111 405–414. 10.1152/jn.00322.2013 24155008 PMC3921387

[B51] Perez M. A. Cohen L. G. (2009). Interhemispheric inhibition between primary motor cortices: what have we learned? *J. Physiol.* 587(Pt 4), 725–726. 10.1113/jphysiol.2008.166926 19103676 PMC2669965

[B52] Petitjean M. Ko J. Y. (2013). An age-related change in the ipsilateral silent period of a small hand muscle. *Clin Neurophysiol* 124 346–353. 10.1016/j.clinph.2012.07.006 22883478

[B53] Plow E. B. Cunningham D. A. Bonnett C. Gohar D. Bayram M. Wyant A. (2013). Neurophysiological correlates of aging-related muscle weakness. *J. Neurophysiol.* 110 2563–2573. 10.1152/jn.00205.2013 24027104 PMC3882769

[B54] Rossetti H. C. Lacritz L. H. Cullum C. M. Weiner M. F. (2011). Normative data for the Montreal Cognitive Assessment (MoCA) in a population-based sample. *Neurology* 77 1272–1275. 10.1212/WNL.0b013e318230208a 21917776

[B55] Rossini P. M. Barker A. T. Berardelli A. Caramia M. D. Caruso G. Cracco R. Q. (1994). Non-invasive electrical and magnetic stimulation of the brain, spinal cord and roots: basic principles and procedures for routine clinical application. *Report of an IFCN committee*. *Electroencephalogr. Clin. Neurophysiol.* 91 79–92. 10.1016/0013-4694(94)90029-9 7519144

[B56] Rothwell J. C. Hallett M. Berardelli A. Eisen A. Rossini P. Paulus W. (1999). Magnetic stimulation: motor evoked potentials. The International Federation of Clinical Neurophysiology. *Electroencephalogr. Clin. Neurophysiol. Suppl.* 52 97–103. 10590980

[B57] Rothwell J. C. Higuchi K. Obeso J. A. (1998). The offset cortical potential: an electrical correlate of movement inhibition in man. *Mov. Disord.* 13 330–335. 10.1002/mds.870130221 9539349

[B58] Ruddy K. L. Leemans A. Carson R. G. (2017). Transcallosal connectivity of the human cortical motor network. *Brain Struct. Funct.* 222 1243–1252. 10.1007/s00429-016-1274-1 27469272 PMC5368198

[B59] Sebastjan A. Skrzek A. Ignasiak Z. Slawinska T. (2017). Age-related changes in hand dominance and functional asymmetry in older adults. *PLoS One* 12:e0177845. 10.1371/journal.pone.0177845 28558047 PMC5448747

[B60] Silbert L. C. Nelson C. Holman S. Eaton R. Oken B. S. Lou J. S. (2006). Cortical excitability and age-related volumetric MRI changes. *Clin. Neurophysiol.* 117 1029–1036. 10.1016/J.Clinph.2006.02.003 16564739

[B61] Stoll T. Huber E. Seifert B. Michel B. A. Stucki G. (2000). Maximal isometric muscle strength: normative values and gender-specific relation to age. *Clin. Rheumatol.* 19 105–113. 10.1007/s100670050026 10791620

[B62] Talelli P. Ewas A. Waddingham W. Rothwell J. C. Ward N. S. (2008a). Neural correlates of age-related changes in cortical neurophysiology. *Neuroimage* 40 1772–1781. 10.1016/j.neuroimage.2008.01.039 18329904 PMC3715371

[B63] Talelli P. Waddingham W. Ewas A. Rothwell J. C. Ward N. S. (2008b). The effect of age on task-related modulation of interhemispheric balance. *Exp. Brain Res.* 186 59–66. 10.1007/s00221-007-1205-8 18040671 PMC2257995

[B64] Terada K. Ikeda A. Nagamine T. Shibasaki H. (1995). Movement-related cortical potentials associated with voluntary muscle relaxation. *Electroencephalogr. Clin. Neurophysiol.* 95 335–345. 10.1016/0013-4694(95)00098-j 7489662

[B65] Woytowicz E. Whitall J. Westlake K. P. (2016). Age-related changes in bilateral upper extremity coordination. *Curr. Geriatr. Rep.* 5 191–199. 10.1007/s13670-016-0184-7 27917365 PMC5130310

