# Supplementary material for: Contraction Phase and Force Differentially Change Motor Evoked Potential Recruitment Slope and Interhemispheric Inhibition in Young Versus Old
Source: Front Hum Neurosci. 2020 Oct 6;14:581008. doi: 10.3389/fnhum.2020.581008 (PMC7573560; doi:10.3389/fnhum.2020.581008)
Supplement: Supplementary file 1 [file Data_Sheet_1.docx]

**Supplementary Material**

# **Supplementary Data**

In order to compare our novel recruitment curve method to a more traditional resting motor threshold (rMT) interval-based method, we collected TMS data on n=10 participants (n=6 younger, n= 4 older) using both methods. Participants provided written informed consent to participate, and they met the same eligibility criteria documented in section “Participants” of the manuscript. Ten samples were collected at each of eight intensities across two task phases (Rest and Execution) and the two recruitment curve methods. Order of task phase and recruitment curve method were counterbalanced across participants. TMS intensities for our method (“anatomical”) are detailed in section “MEP Recruitment Sampling”. For the traditional method, TMS intensities were given in 10% rMT intervals from 90% to 160% rMT, in semi-random order. As shown in Supplementary Figure 1, our method did achieve its goal of sampling the most linear aspect of the recruitment curve and was more efficient than trying to find the plateau level of motor evoked potential (MEP) amplitude for each participant.


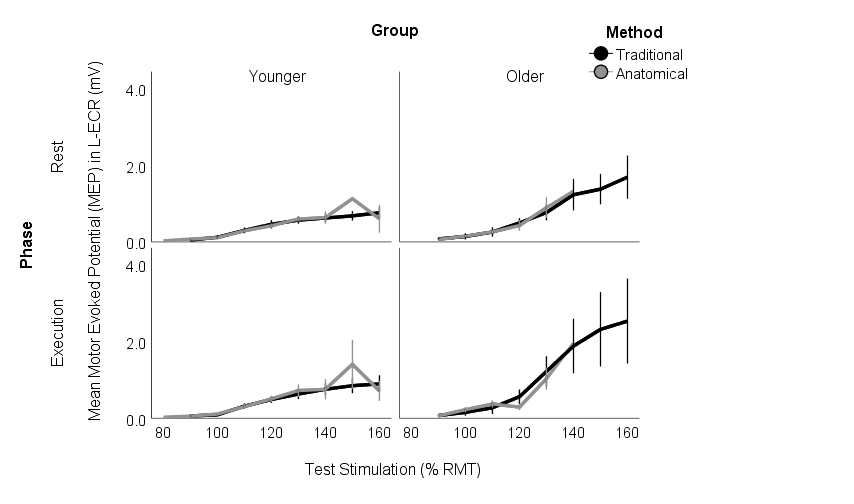


Supplementary Figure 1. Mean peak-to-peak motor-evoked potential in the L-ECR as a function of percent of resting motor threshold across task phases and groups. Data from a sample (n=10) comparing the novel method (“anatomical”) used in this manuscript and the traditional method of recruitment curve sampling.
